# Effect of aromatherapy on autonomic nervous system regulation with treadmill exercise-induced stress among adolescents

**DOI:** 10.1371/journal.pone.0249795

**Published:** 2021-04-13

**Authors:** Pin-Hsuan Lin, Yuan-Ping Lin, Kai-Li Chen, Shang-Yu Yang, Yin-Hwa Shih, Po-Yu Wang

**Affiliations:** 1 Department of Health and Beauty, Shu Zen Junior College of Medicine and Management, Kaohsiung, Taiwan; 2 Department of Nursing, College of Pharmacy and Health Care, Tajen University, Pingtung, Taiwan; 3 Department of Healthcare Administration, College of Medical and Health Science, Asia University, Taichung, Taiwan; 4 Department of Pediatric Emergency, Changhua Christian Children Hospital, Changhua, Taiwan; University of Ljubljana, Medical faculty, SLOVENIA

## Abstract

**Introduction:**

Stress is a major health issue in adolescents owing to the important transitions experienced during this period. Aromatherapy is an effective method for the reduction of stress in adolescents.

**Purpose:**

The aims of this study were to examine the effect of aromatherapy on the regulation of the autonomic nervous system (ANS) along with stress relief and to explore the effect of aromatherapy on adolescents with different levels of stress.

**Methods:**

This quasi-experimental study comprised three types of treatments: control (no essential oil), pure essential oil therapy (sandalwood), and blended essential oil therapy (sandalwood-lavender). The heart rate variability (HRV) was calculated to evaluate the post-exercise recovery of the ANS to the baseline level in the recruited adolescents. To examine the efficiency of aromatherapy, Friedman test was used to assess the significance of difference in all parameters (i.e., mean heart rate, SDNN, normalized LF, normalized HF, and LF/HF) between baseline and after exercise among the three treatment conditions.

**Results:**

The participants comprised 43 junior college students (8 males and 35 females) with a mean age of 18.21 ± 0.99. Significant differences in changes of two HRV parameters (normalized LF and LF/HF) were associated with both essential oil therapies compared to those in the control group (*p*<0.05), and one more HRV parameter (normalized HF) exhibited significant difference related to blended essential oil therapy compared to that of the control group. Besides, changes in two HRV parameters (mean heart rate and normalized HF) of both essential oil therapies in the low level stress subgroup showed significant differences compared to those of the control group (*p*<0.05).

**Conclusions:**

This study demonstrated that aromatherapy could be used for ANS regulation with stress-relieving effects in adolescents. The participants with a low stress level appeared to respond better to the blended essential oil therapy, whereas those with medium to high levels of stress appeared to respond poorly to aromatherapy compared to the control.

## Introduction

Stress may have short-term and long-term negative consequences for the emotional and physical health of adolescents [1]. Additionally, the demands of the social environment, such as changes in the relationships with parents, teachers and peers [2,3], high expectations and pressure from the family, and the high demand in academic performance at school burden the adolescents with additional physical and mental stress [4–8]. Unfortunately, many adolescents are unable to cope with the stress they encounter in the modern era [9]. Because most of the stress comes from the unavoidable external environment related to studies [5,10,11] or schooling [12], it is difficult to relieve these stresses effectively.

The use of biological means to reduce stress through modulation of the autonomic nervous system (ANS) has been suggested [13]. The ANS, which includes the sympathetic nervous system (SNS) and the parasympathetic nervous system (PNS), is crucial for the physical response to stress [14]. Stress is reflected by changes in the activities of the SNS and the PNS, which can alter the breathing pattern in conjunction with the heart rate. Heart rate variability (HRV), which is a measure of the variation in heartbeats within a specific timeframe acquired through electrocardiogram, impedance-plethysmography or photo-plethysmography [15], has been widely used as a non-invasive means to assess the functional balance between the SNS and PNS [16]. Because HRV analysis can provide the physiological signals for stress assessment in the clinical setting [17], it has been established as a reliable and popular tool to assess the status of cardiovascular autonomic function [18].

A recent systematic review has summarized the evidence supporting the effectiveness of essential oil aromatherapy for stress reduction in adolescents across various countries [19]. Essential oils can be absorbed into the body through the digestive system, skin, or olfactory system with the response being fastest via the olfactory system [20,21]. Previous reports have demonstrated that absorption of essential oils through 15 min inhalation can affect the ANS system and improve the physical, mental, and emotional well-being [19,20]. However, most previous studies were conducted in specific populations such as nurses [22] or patients [23,24]. In one study, aromatherapy was found to be effective for relieving stress in 36 high-school female students [25]. Nevertheless, information about the effect of aromatherapy in the adolescent population is still insufficient.

HRV has been successfully used to evaluate ANS functional recovery in young individuals following exercise stress stimulation (i.e., treadmill test) [26], which is a common approach to evaluating the cardiac response to stress [27]. Although sympathetic activation is typically reflected by the symptoms of stress (i.e., heart rate > 100 beats per minute with a rapid increase in respiratory rate) [28], ANS activities may vary with the levels of stress in different individuals [29]. In the current study, treadmill-induced sympathetic activation to a heart rate of over 100 per minute was used to investigate the effect of aromatherapy on ANS regulation. The aims of this study were to examine the effect of aromatherapy on ANS regulation and stress relief in adolescents with different levels of stress. The findings of this study could provide more information about the role of aromatherapy in maintaining ANS balance and achieving stress reduction in adolescents.

## Methods

### Participants

Eligible adolescents were recruited from a junior college in Taiwan between August and November 2017. The participants were recruited on a voluntary basis after being given full explanation regarding the purpose and the inclusion/exclusion criteria of the study by the research assistants of the study. All participants were required to provide informed written consents before being given the questionnaires for the current study. For participants younger than 18 years, consents from their parents or guardians were needed. All participants met the following inclusion criteria: no clinical diagnoses of respiratory disorders, hypertension, and heart disease and no history of asthma and/or allergies to flowers, plants, and essential oils. The exclusion criteria were participants with a clinical diagnosis of mental illness and/or those who did not complete the baseline questionnaire. Ethical approval for this study was obtained from the National Cheng Kung University Human Research Ethics Committee (NCKU HREC-E-106-108-2). All procedures were performed in accordance with the relevant guidelines and regulations.

### Study design and procedure

This quasi-experimental study comprised three treatments: a control treatment (no essential oil) and two experimental treatments using either pure essential oil (i.e., sandalwood) or blended essential oil (i.e., sandalwood-lavender). The participants were required to undergo the control, pure, and blended essential oil therapy sessions in turns with a 48-hour washout period using a cross-over design to eliminate any order effect. Each participant was consecutively assigned into six blocks in the following sequence: ABC, ACB, BAC, BCA, CAB, and CBA, wherein A denoted the control session, B the pure oil session and C the blended essential oil session. The cycle was then repeated after each session for all participants.

**Fig 1:**
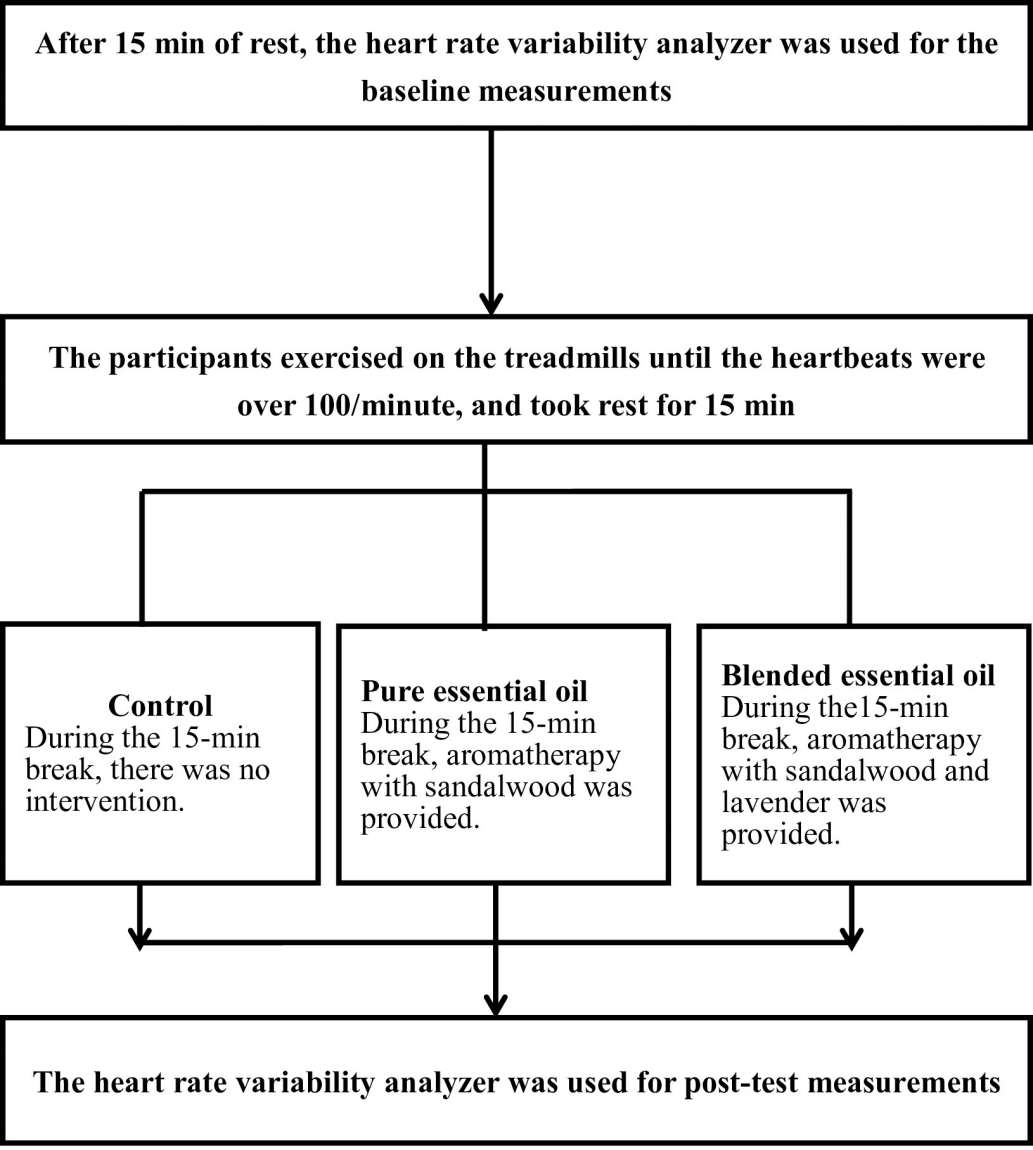
Flowchart of the experiments.

The experimental flow chart is shown in Fig 1. All participants were first asked to rest on a chair for 15 min, followed by an assessment of their baseline ANS performance with an HRV analyzer in a sitting and relaxed position with the eyes open. The participants were then required to walk on the treadmill (zero degree inclination) with a structured increase in walking speed until reaching and holding required as well as a heartbeat rate over 100/min for 10 seconds. The participants were allowed to rest for 15 min in the same position as they did during their baseline assessment. During this resting period, the participants in the control session did not receive any intervention, whereas those attending the pure and blended essential oil treatment sessions received aromatherapy. Subsequently, the HRV analyzer was used to obtain the post-test (sitting, eye open, and no activity) measurements. All activities were conducted in the same quiet, temperature- and humidity-controlled room (approximately 33 square meters; temperature, 24°C–26°C; humidity, 50%–65%).

### Questionnaire

The questionnaire used in this study contained demographic and anthropometric information (i.e., sex, age, body weight and height, body mass index [BMI], smoking [yes = smoking ≥ 1 cigarette/day for the past 6 months] and drinking habits [yes = drinking alcoholic beverages ≥ once/week for the past 6 months] as well as the Stress Index Scale. The Stress Index Scale designed by the Taiwan Health Promotion Administration comprises 12 questions with "yes" (1 point) and "no" (0 point) answers and a score ranging from 0 to 12 [30]. The higher the score, the greater the stress: Score 0–3 indicates light stress; 4–5 medium stress; 6–8 high stress; 9–12 very high stress [30]. In the current study, the Cronbach’s alpha coefficient for the overall Stress Index Scale score was 0.78.

### Aromatherapy intervention

After being diluted 1:75 with distilled water, the mist of the essential oil was dispensed through an ultrasonic aromatherapy diffuser (type YHL699, ultrasound frequency 2.5 MHz, Nature Creart Co., Ltd., Taiwan) which was placed approximately 50 cm away from the participants. The pure essential oil treatment comprised 2.5% sandalwood (Botanical name: Santalum spicatum, Chris Botanica Co. Ltd, Taichung, Taiwan), while the blended essential oil treatment consisted of 2.5% sandalwood and 2.5% lavender (Botanical name: Santalum spicatum and Lavendula angustifolia, Chris Botanica Co. Ltd, Taichung, Taiwan) inhalation during a 15 min break after the treadmill exercise. The participants who attended the control session received no intervention during the break after the physical exercise. The activity of ANS was assessed using the HRV SA-3000p analyzer (Medicore Co., Ltd, Seoul, Korea) before exercise (baseline measurement) and during the break (post-test measurement).

### HRV analysis

Five HRV parameters were collected as follows: (1) Mean heart rate: Average heart beat (beats per minute, BPM); (2) SDNN: The standard deviation of all the normal-to-normal intervals (SDNN) for time domain analysis, which is defined as the standard deviation of the RR interval sequence, is a standard parameter of the overall HRV that is influenced by both sympathetic and parasympathetic nervous activities. It represents the activation status of the ANS [31] and may be significantly reduced when an individual is overworked, stressed, or sick (particularly in diabetic patients) [32,33]; (3) Low frequency band (LF): It signifies the result of a mixed modulation of sympathetic and parasympathetic activities. The power of the LF was computed in the range of 0.04–0.15 Hz, and the normalized values of LF (i.e., normalized LF) were calculated for statistical analysis; (4) High frequency band (HF): It reflects the parasympathetic nervous activity. The power of the HF was computed in the range of 0.15–0.4Hz, and the normalized values of HF (i.e., normalized HF) were calculated for statistical analysis; (5) LF/HF: The ratio of LF/HF denotes the sympathetic nervous activity relative to that of the parasympathetic nervous system. A ratio of "1" implies similar activities between the sympathetic and parasympathetic nerves, whereas the activation of the sympathetic system is higher than that of the parasympathetic system if the ratio exceeds 1.

### Statistical analysis

Descriptive statistics were used to analyze the demographic variables, including sex, age, BMI, smoking and drinking habits as well as the Stress Index Scale. To examine the efficiency of aromatherapy, Friedman test was adopted to analyze the absolute differences (i.e., “after intervention” minus “baseline”) of non-normally distributed variables (i.e., mean heart rate, SDNN, normalized LF, normalized HF, and LF/HF) to determine the significance of difference among the three treatment conditions (i.e., control, pure oil, and blended oil). Then, the Wilcoxon signed-rank test was used to determine the significance of the difference between control treatment and two experimental treatments when Friedman tests were significant. Moreover, the participants were divided into two subgroups for examining the efficiency of the aromatherapy among those with different levels of stress. Based on the scores of Stress Index Scale, the participants were divided into those with low, medium, and high levels of stress. All calculations were performed using the IBM SPSS Statistics version 22 for Mac (IBM Corp., Armonk, NY). The significance threshold was set at *P* < 0.05. Sample size was estimated using G-power software (3.1.0). With an alpha level of 0.05, a power of 80%, and an expected effect size of 0.5, a total sample size of 35 individuals would be required according to the estimation provided by the Wilcoxon signed-rank test for matched pairs in G*Power [34].

## Results

### Demography of the participants

Junior college students (N = 43; 8 males and 35 females) with a mean age of 18.21 ± 0.99 (standard deviation) years (range, 16–20 years) were recruited with no participant being excluded due to due to mental illness or questionnaire incompleteness. The mean BMI was 22.65 ± 4.27 (range, 17.7–38.5); 12 students (27.9%) were overweight (i.e., BMI ≥ 24). There were 27 (62.8%), 14 (32.6%), and 2 (4.7%) participants presenting with low, medium, and high levels of stress, respectively, with the mean score of the Stress Index Scale being 3.3 ± 1.2. None of the participants reported smoking or drinking habits. In addition, the means and standard deviations of HRV parameters associated with the control, pure essential oil (sandalwood), and blended essential oil (sandalwood and lavender) treatments before and after exercise with or without intervention among adolescents with different levels of stress are shown in S1-S3 Appendices.

### Changes in HRV parameters for the three treatments

**Fig 2:**
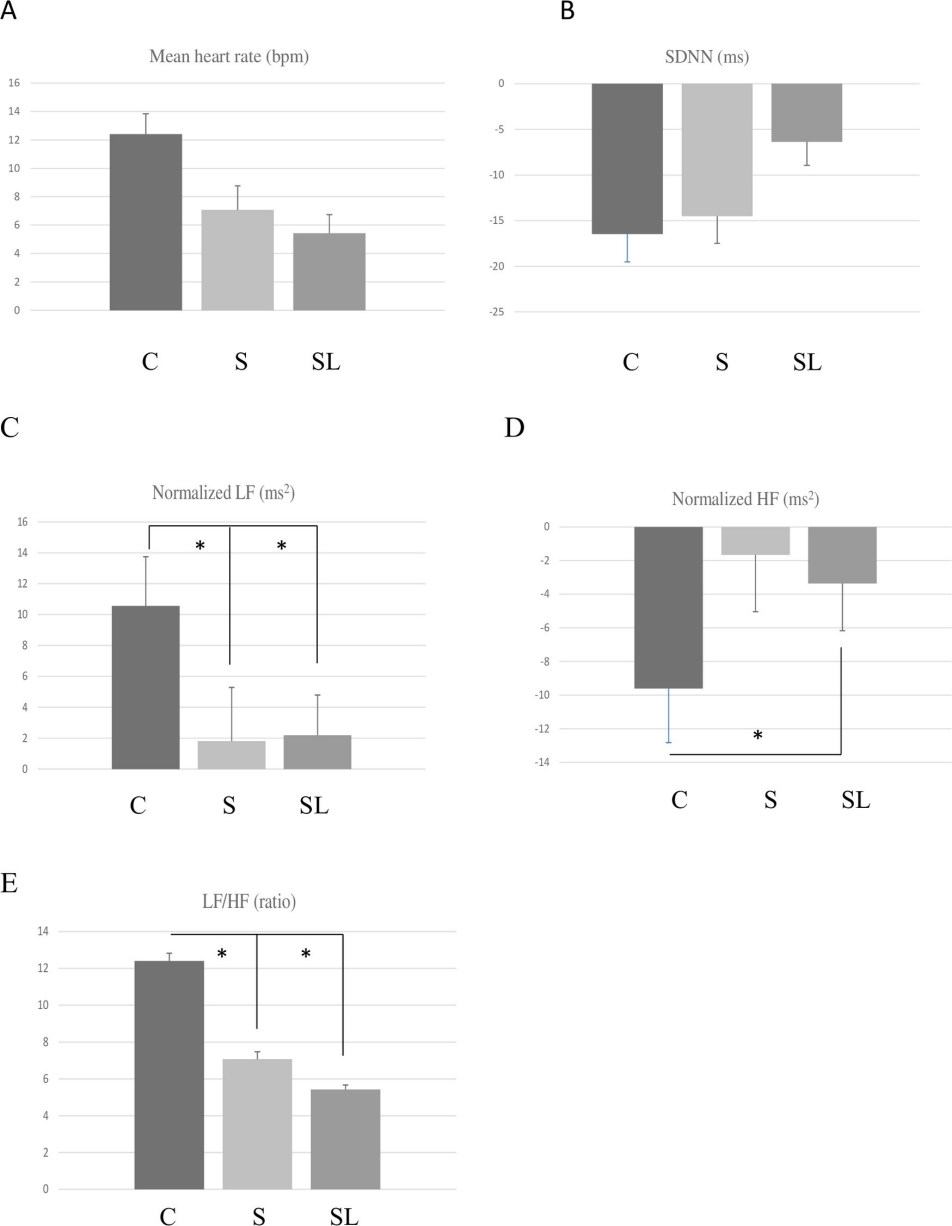
Comparison of the HRV parameters among the control (C), pure essential oil (sandalwood, S), and blended essential oil (sandalwood and lavender, SL) treatments (Mean ± SE; * *P* < 0.05). (A) Mean heart rate; (B) SDNN; (C) Normalized LF; (D) Normalized HF; (E) Normalized HF.

A comparison of the post-exercise changes in HRV parameters among the control, pure essential oil, and blended essential oil treatments is shown in Table 1 and Fig 2. Friedman test showed significant differences in the changes of three HRV parameters (i.e., normalized LF, normalized HF, and LF/HF) among the three treatments. The Wilcoxon signed-rank test demonstrated significant differences in the changes of two HRV parameters (i.e., normalized LF, and LF/HF) between the control and pure essential oil treatment, while the changes in three HRV parameters (i.e., normalized LF, normalized HF, and LF/HF) were significantly different between the control and blended essential oil treatment.

**Table 1:** Comparison of post-exercise changes in HRV parameters among the control, pure essential oil (sandalwood), and blended essential oil (lavender essential oil) treatments (N = 43).

HRV parameters	Control (C)		Sandalwood (S)		Sandalwood and lavender (SL)		*p*-value (Wilcoxon signed-rank test)
	mean ± standard deviation	median value (interquartile range)	mean ± standard deviation	median value (interquartile range)	mean ± standard deviation	median value (interquartile range)	
**Mean heart rate (bpm)**	12.40 ± 9.45	11.0 (6.0–18.0)	7.07 ± 11.16	10.0 (3.0–14.0)	5.42 ± 8.62	6.0 (0.0–12.0)	0.09
**SDNN (ms)**	-16.46 ± 20.05	-16.2 (-28.4- -4.2)	-14.51 ± 19.46	-12.7 (-31.9–3.3)	-6.37 ± 16.77	-9.6 (-15.8–2.7)	0.20
**Normalized LF (ms^2^)**	10.56 ± 20.95	12.0 (-1.0–24.0)	1.81 ± 22.84	2.0 (-9.0–17.0)	2.19 ± 17.10	2.0 (-11.0–12.0)	0.02* (C)>(S)*; (C)>(SL)*
**Normalized HF (ms^2^)**	-9.60 ± 21.17	-12.0 (-24.0–1.0)	-1.67 ± 22.15	-2.0 (-17.0–9.0)	-3.37 ± 18.34	-2.0 (-14.0–11.0)	0.05* (C)<(SL)*
**LF/HF (ratio)**	1.50 ± 2.81	1.0 (-0.1–2.8)	0.31 ± 2.65	-0.0 (-0.9–2.3)	0.10 ± 1.67	0.1 (-1.0–1.1)	0.05* (C)>(S)*; (C)>(SL)*

HRV: Heart rate variability; SDNN: Standard deviation of all RR intervals; LF: Low frequency; HF: High frequency; bpm: Beats per minute.

*P < 0.05.

### HRV parameters for the three treatments with different levels of stress

Comparisons of the post-exercise changes in HRV parameters (mean ± SD) among the control, pure essential oil, and blended essential oil treatments based on the levels of stress are shown in Tables 2 and 3. For the subgroup with a low level of stress, Friedman test revealed significant changes in three HRV parameters (i.e., mean heart rate, normalized LF, and normalized HF) among the three treatments. The Wilcoxon signed-rank test showed significant differences in the changes of mean heart rate and normalized HF between the control and pure essential oil treatments (both *P* < 0.05), whereas significant differences in the changes of mean heart rate, normalized LF and normalized HF were observed between the control and blended essential oil treatment (all *P* < 0.05) (Table 2). For the subgroups with medium and high levels of stress, no significant differences in the changes of any of the HRV parameters were noted among the three treatments (Table 3). The participants with a low stress level appeared to respond better to the blended essential oil aromatherapy, whereas those with medium to high levels of stress appeared to respond poorly to aromatherapy compared to the control.

**Table 2:** Comparison of post-exercise changes in HRV parameters among the control, pure essential oil (sandalwood), and blended essential oil (sandalwood and lavender) treatments in participants with a low stress level (n = 27).

HRV parameters	Control (C)		Sandalwood (S)		Sandalwood and lavender (SL)		*p*-value (Wilcoxon signed-rank test)
	mean ± standard deviation	median value (interquartile range)	mean ± standard deviation	median value (interquartile range)	mean ± standard deviation	median value (interquartile range)	
**Mean heart rate (bpm)**	14.44 ± 10.33	11.0 (7.0–19.0)	7.11 ± 11.79	9.0 (3.0–14.0)	5.52 ± 8.23	5.0 (1.0–12.0)	0.01** (C)>(S)**; (C)>(SL)**
**SDNN (ms)**	-17.68 ± 21.06	-18.3 (-31.5- -3.9)	-16.06 ± 21.08	-12.8 (-32.3–1.7)	-5.60 ± 16.45	-9.6 (-15.8–2.7)	0.10
**Normalized LF (ms^2^)**	14.15 ± 21.63	18.0 (1.0–31.0)	3.70 ± 23.53	2.0 (-9.0–17.0)	1.81 ± 18.39	2.0 (-12.0–11.0)	0.03* (C)>(SL)**
**Normalized HF (ms^2^)**	-13.96 ± 21.43	-18.0 (-29.0- -1.0)	-3.56 ± 22.45	-2.0 (-17.0–9.0)	-1.85 ± 18.41	-2.0 (-11.0–12.0)	0.03* (C)<(S)*; (C)<(SL)*
**LF/HF (ratio)**	1.53 ± 2.82	1.1 (-0.1–2.8)	0.49 ± 2.59	-0.0 (-0.9–2.8)	0.22 ± 1.66	0.1 (-0.8–1.1)	0.22

HRV: Heart rate variability; SDNN: Standard deviation of all RR intervals; LF: Low frequency; HF: High frequency; bpm: Beats per minute.

*P < 0.05

**P < 0.01.

**Table 3:** Comparison of post-exercise changes in HRV parameters among the control, pure essential oil (sandalwood), and blended essential oil (sandalwood and lavender) treatments in participants with medium to high levels of stress (n = 16).

HRV parameters	Control (C)		Sandalwood (S)		Sandalwood and lavender (SL)		*p*-value
	mean ± standard deviation	median value (interquartile range)	mean ± standard deviation	median value (interquartile range)	mean ± standard deviation	median value (interquartile range)	
**Mean heart rate (bpm)**	8.94 ± 6.71	8.0 (3.5–14.3)	7.00 ± 10.40	10.0 (4.3–14.8)	5.25 ± 9.52	7.0 (0.0–11.8)	0.81
**SDNN (ms)**	-14.41 ± 18.71	-14.4 (-22.1- -4.3)	-11.89 ± 16.69	-12.1 (-27.2–6.3)	-7.66 ± 17.77	-9.5 (-21.8–1.8)	0.81
**Normalized LF (ms^2^)**	4.50 ± 18.85	3.0 (-6.3–18.5)	-1.38 ± 22.00	1.0 (-15.0–16.8)	2.81 ± 15.22	2.0 (-6.3–14.3)	0.52
**Normalized HF (ms^2^)**	-2.25 ± 19.15	-1.0 (-16.0–8.5)	1.50 ± 21.97	-1.0 (-16.8–15.0)	-5.94 ± 18.51	-5.0 (-20.3–6.3)	0.56
**LF/HF (ratio)**	1.44 ± 2.88	0.1 (-0.5–3.6)	0.01 ± 2.81	0.1 (-0.9–1.7)	-0.12 ± 1.72	0.2 (-1.4–1.1)	0.21

HRV: Heart rate variability; SDNN: Standard deviation of all RR intervals; LF: Low frequency; HF: High frequency; bpm: Beats per minute.

## Discussion

The current study, which assessed the therapeutic effect of aromatherapy against stress in adolescents through ANS modulation, focused on stress relaxation in an acute rather than a chronic setting. Moreover, whether similar effects can be observed in individuals with high levels of psychological stress remains unclear. The three treatments were provided for the same batch of participants by using a cross-over design to eliminate the influence of demographic factors and order effects on the results. Therefore, the decrease in stress (i.e., activation of PNS) after experimental treatment could be attributed to the effect of the aromatherapy.

Two forms of essential oils are commonly used for aromatherapy, namely pure essential oil (i.e., sandalwood) or blended essential oil (i.e., sandalwood-lavender). Both sandalwood and sandalwood-lavender essential oils, which are widely used in aromatherapy to reduce stress or physical discomfort such as pain and sleep disorders [35–38], have been shown to reduce anxiety and stress in women undergoing breast biopsy [38] and patients receiving palliative care [39]. However, the effectiveness of these two essential oils for stress relief in the adolescent population remains unclear. Our results showed that blended essential oil (i.e., sandalwood and lavender) was effective on the regulation of the autonomic nervous system (ANS) along with stress relief (Table 1). The effect of blended essential oil treatment on normalized LF, normalized HF and LF/HF (ratio) was accompanied by a decrease in sympathetic nervous activity and an activation of the parasympathetic nerves. The minor post-exercise fluctuation in normalized LF associated with blended essential oil treatment compared with that in the control may indicate a reduction in sympathetic tone and stress relief following blended essential oil aromatherapy. Moreover, the blended essential oil aromatherapy also increased the HF parameter and reduced the LF/HF ratio, implying that this treatment may relieve emotional stress within a short time because the HF parameter is related to emotional processing [40]. Our findings was in line with the those of a previous study [13].

In addition, several studies [41,42] have confirmed the effectiveness of aromatherapy for reducing stress. In the current study, we explored the effect of aromatherapy on ANS activities of the participants with different levels of stress and observed that aromatherapy may be more suitable for individuals with a low level of stress or anxiety. However, the finding was not in line with that of a previous study [13], in which patients with a high degree of anxiety benefited more from aromatherapy than those with mild anxiety. This may be attributed to the differences in the sources of stress, concentrations of essential oil, and populations between the two studies. Nevertheless, additional studies on the effectiveness of aromatherapy in people with different degrees of anxiety/stress are warranted. Compared with pure essential oil treatment, the present study demonstrated that blended essential oil aromatherapy was associated with significant change in one more HRV parameter (Table 2); not only did blended essential oil aromatherapy reduce the mean heart rate and LF/HF ratio but it also increased the HF parameter, suggesting its effectiveness for relieving emotional stress.

Furthermore, compared with pure essential oil treatment, blended essential oil treatment was associated with significant changes in more HRV parameters after exercise in all participants and those with a low stress level in the current study. Therefore, the subjects who received blended essential oil treatment seemed to respond more favorably compared to those undergoing pure essential oil treatment. Previous studies have shown that both sandalwood and lavender are most commonly used in the clinical setting because of their ability to promote a feeling of relaxation, calmness, and sedation [38,39,43]. The sandalwood essential oil consists of α- and β-santalols, which contribute to its reputed sedative effect [44], whereas the lavender essential oil comprises β-linalool, β-caryophyllene, and linalyl acetate, which act as a muscle relaxant, antidepressant, and sedative [45]. Moreover, a recent randomized controlled trial has provided evidence to support blended essential oil (sandalwood and lavender) treatment to minimize stress in humans [38], possibly attributable to the combined advantages of its two pure essential oil components.

Although we tried to reduce the influence of demographics and order effects on the results of the present study, some limitations need to be taken into account for correct interpretation of its findings. First, although a heart rate of over 100/min in subjects undergoing a treadmill test is seen as a physical sign of stress, such a simulation may not reflect the psychological stress of an individual. Moreover, differences in the time taken to achieve a heart rate of 100 beats/min could be a confounder. Second, because the sample size was small and the participants were all from a single college in Taiwan, future studies on a larger population with wider geographical and ethnical coverage are needed to support our findings. Third, HRV may be influenced by the concentration of the essential oils, inhalation time, environmental temperature and humidity as well as personal responses to aromatherapy. Fourth, because this was not a randomized controlled study, high-quality causal evidence cannot be generated.

## Conclusion

Aromatherapy with sandalwood and lavender essential oils through 15 min inhalation after exercise stimulation could enhance parasympathetic activity in adolescents, for whom blended essential oil (sandalwood and lavender) was effective on the regulation of the ANS along with stress relief. Therefore, this study demonstrated that aromatherapy may be used for ANS regulation with stress-relieving effects in adolescents.

## Supporting information

S1 Appendix The mean and standard deviation of HRV parameters among the control, pure essential oil (sandalwood), and blended essential oil (lavender essential oil) treatments before and after the intervention. (DOCX)

S2 Appendix The mean and standard deviation of HRV parameters among the control, pure essential oil (sandalwood), and blended essential oil (sandalwood and lavender) treatments before and after intervention in participants with light levels of stress. (DOCX)

S3 Appendix The mean and standard deviation of HRV parameters among the control, pure essential oil (sandalwood), and blended essential oil (sandalwood and lavender) treatments before and after intervention in participants with medium to high levels of stress. (DOCX)

S1 Dataset (SAV)
