# Supplementary material for: Effect of aromatherapy on autonomic nervous system regulation with treadmill exercise-induced stress among adolescents
Source: PLoS One. 2021 Apr 13;16(4):e0249795. doi: 10.1371/journal.pone.0249795 (PMC8043395; doi:10.1371/journal.pone.0249795)
Supplement: S1 Appendix — (DOCX) [file pone.0249795.s001.docx]

**Appendix 1. The mean and standard deviation of HRV parameters among the control, pure essential oil (sandalwood), and blended essential oil (lavender essential oil) treatments before and after the intervention.**

| HRV parameter | n = 43 | | |
| --- | --- | --- | --- |
|  | Control (C) | Sandalwood (S) | Sandalwood and lavender (SL) |
| Mean heart rate (bpm) |  |  |  |
| Baseline | 81.26 ± 9.53 | 80.54 ± 11.81 | 82.16 ± 11.11 |
| After | 93.65 ± 11.14 | 87.61 ± 13.65 | 87.58 ± 12.80 |
| SDNN (ms) |  |  |  |
| Baseline | 48.65 ± 19.27 | 52.02 ± 19.76 | 48.55 ± 15.58 |
| After | 32.18 ± 15.44 | 37.55 ± 12.57 | 42.25 ± 18.75 |
| LF (ms^2^) |  |  |  |
| Baseline | 642.18 ± 695.15 | 870.05 ± 906.91 | 769.24 ± 870.28 |
| After | 341.62 ± 405.96 | 467.41 ± 465.76 | 469.88 ± 469.61 |
| HF (ms^2^) |  |  |  |
| Baseline | 445.48 ± 479.16 | 497.38 ± 482.79 | 408.21 ± 290.21 |
| After | 145.97 ± 160.93 | 222.05 ± 179.73 | 260.65 ± 221.92 |
| Normalized LF |  |  |  |
| Baseline | 56.92 ± 18.35 | 63.24 ± 19.13 | 59.86 ± 17.03 |
| After | 67.39 ± 19.49 | 65.01 ± 19.97 | 62.00 ± 14.28 |
| Normalized HF |  |  |  |
| Baseline | 42.12 ± 19.29 | 36.89 ± 19.31 | 39.95 ± 17.14 |
| After | 32.60 ± 19.49 | 35.28 ± 19.64 | 36.61 ± 14.15 |
| LF/HF (ratio) |  |  |  |
| Baseline | 1.95 ± 1.43 | 2.66 ± 2.10 | 2.03 ± 1.50 |
| After | 3.69 ± 3.90 | 2.97 ± 2.81 | 2.12 ± 1.38 |

HRV: heart rate variability; SDNN: standard deviation of all RR intervals; LF: low frequency; HF: high frequency.
