# Supplementary material for: Effect of aromatherapy on autonomic nervous system regulation with treadmill exercise-induced stress among adolescents
Source: PLoS One. 2021 Apr 13;16(4):e0249795. doi: 10.1371/journal.pone.0249795 (PMC8043395; doi:10.1371/journal.pone.0249795)
Supplement: S3 Appendix — (DOCX) [file pone.0249795.s003.docx]

**Appendix 3. The mean and standard deviation of HRV parameters among the control, pure essential oil (sandalwood), and blended essential oil (sandalwood and lavender) treatments before and after intervention in participants with medium to high levels of stress.**

| HRV parameter | n = 16 | | |
| --- | --- | --- | --- |
|  | Control (C) | Sandalwood (S) | Sandalwood and lavender (SL) |
| Mean heart rate (bpm) |  |  |  |
| Baseline | 81.75 ± 8.65 | 80.69 ± 6.32 | 83.94 ± 6.95 |
| After | 90.69 ± 9.46 | 87.69 ± 12.14 | 89.19 ± 9.93 |
| SDNN (ms) |  |  |  |
| Baseline | 51.22 ± 19.91 | 47.71 ± 15.63 | 46.66 ± 18.52 |
| After | 36.86 ± 17.36 | 35.93 ± 8.78 | 39.10 ± 19.28 |
| LF (ms^2^) |  |  |  |
| Baseline | 850.24 ± 886.43 | 778.59 ± 574.37 | 819.87 ± 1110.41 |
| After | 436.41 ± 444.40 | 381.84 ± 287.90 | 474.29 ± 500.04 |
| HF (ms^2^) |  |  |  |
| Baseline | 515.55 ± 692.06 | 405.60 ± 322.75 | 334.16 ± 201.12 |
| After | 186.92 ± 195.26 | 192.33 ± 180.56 | 220.35 ± 204.94 |
| Normalized LF |  |  |  |
| Baseline | 61.00 ± 16.04 | 67.31 ± 14.52 | 59.83 ± 20.76 |
| After | 65.41 ± 20.34 | 65.90 ± 19.89 | 62.63 ± 17.45 |
| Normalized HF |  |  |  |
| Baseline | 36.71 ± 18.70 | 32.69 ± 14.52 | 40.48 ± 21.11 |
| After | 34.59 ± 20.34 | 34.22 ± 19.74 | 34.57 ± 16.42 |
| LF/HF (ratio) |  |  |  |
| Baseline | 1.96 ± 1.39 | 2.81 ± 2.06 | 2.42 ± 2.06 |
| After | 3.40 ± 3.36 | 2.82 ± 1.89 | 2.30 ± 1.57 |

HRV: heart rate variability; SDNN: standard deviation of all RR intervals; LF: low frequency; HF: high frequency.
